# Promotion of Anatomical Science

**Published:** 1872-04

**Authors:** 


A FRENCH journal says, that a society has been formed in Paris, now numbering more than a hundred members, each of whom declare that it is his wish that his body, after death, be used for the promotion of anatomical science.

---

CHEAP AND DURABLE WROUGHT NAILS.—Every farmer understands the usual method of making cut nails flexible by heating them; but if, instead of allowing them to cool in the open air, they are thrown when red hot into linseed oil, it will prevent their rusting almost as long as if they were galvanized—*Boston Jour. Chemistry.*

